# Coenzyme Q in *Thraustochytrium* sp. RT2316-16: Effect of the Medium Composition

**DOI:** 10.3390/md21110586

**Published:** 2023-11-10

**Authors:** Liset Flores, Carolina Shene, Juan A. Asenjo, Yusuf Chisti

**Affiliations:** 1 Department of Chemical Engineering, Center of Food Biotechnology and Bioseparations, BIOREN, and Centre of Biotechnology and Bioengineering (CeBiB), Universidad de La Frontera, Temuco 4811230, Chile; liset.flores@ufrontera.cl; 2 Centre for Biotechnoloy and Bioengineering (CeBiB), Department of Chemical Engineering, Biotechnology and Materials, Universidad de Chile, Beauchef 851, Santiago 8370459, Chile; juasenjo@ing.uchile.cl; 3 Institute of Tropical Aquaculture and Fisheries, Universiti Malaysia Terengganu, Kuala Nerus 21030, Terengganu, Malaysia; ychisti@hotmail.com

**Keywords:** ubiquinone, thraustochytrids, docosahexaenoic acid, *Thraustochytrium* sp., B vitamins

## Abstract

Coenzyme Q (CoQ; ubiquinone) is an essential component of the respiratory chain. It is also a potent antioxidant that prevents oxidative damage to DNA, biological membranes, and lipoproteins. CoQ comprises a six-carbon ring with polar substituents that interact with electron acceptors and donors, and a hydrophobic polyisoprenoid chain that allows for its localization in cellular membranes. Human CoQ has 10 isoprenoid units (CoQ_10_) within the polyisoprenoid chain. Few microorganisms produce CoQ_10_. This work shows that *Thraustochytrium* sp. RT2316-16 produces CoQ_10_ and CoQ_9_. The CoQ_10_ content in RT2316-16 depended strongly on the composition of the growth medium and the age of the culture, whereas the CoQ_9_ content was less variable probably because it served a different function in the cell. Adding *p*-hydroxybenzoic acid to the culture media positively influenced the CoQ_10_ content of the cell. The absence of some B vitamins and *p*-aminobenzoic acid in the culture medium negatively affected the growth of RT2316-16, but reduced the decline in CoQ_10_ that otherwise occurred during growth. The highest content of CoQ_9_ and CoQ_10_ in the biomass were 855 μg g^−1^ and 10 mg g^−1^, respectively. The results presented here suggest that the thraustochytrid RT2316-16 can be a potential vehicle for producing CoQ_10_. Metabolic signals that trigger the synthesis of CoQ_10_ in RT2316-16 need to be determined for optimizing culture conditions.

## 1. Introduction

Coenzyme Q (CoQ) or ubiquinone is a lipophilic cofactor in the electron transport chain. CoQ comprises a hydrophobic polyisoprenoid chain enabling it to be embedded in cell membranes, and a six-carbon ring with polar substituents allowing the molecule to interact with electron donors and acceptors. In the inner membrane of mitochondria, CoQ transports electrons from complex I (NADH: ubiquinone oxidoreductase, EC 1.6.5.3) or the complex II (succinate: ubiquinone oxidoreductase, EC 1.3.5.1) to complex III (ubiquinol: cytochrome c oxidoreductase, EC 1.10.2.2). In addition to this important function in the generation of chemical energy, CoQ protects the cell against oxidative stress [1,2], helps in regulating mitochondrial permeability transition pores [3], and is involved in mitochondrial homeostasis [4], the biosynthesis of pyrimidines and fatty acid *β*-oxidation [5]. The number of isoprenyl units in the polyisoprenoid chain of CoQ is species-dependent; in humans this number is 10 (CoQ_10_), whereas in the yeast *Saccharomyces cerevisiae* it is 6, and in the bacterium *Escherichia coli* it is 8.

A CoQ_10_ deficiency (a secondary deficiency, i.e., not directly related to genetic mutations [6]) in humans is associated with several diseases and age-related chronic conditions [1,7], linked to high mortality if left untreated. There is some evidence that dietary supplementation with CoQ_10_ improves mitochondrial function and confers antioxidant protection to organs and tissues affected by various pathophysiological conditions [1,7,8]. Therefore, CoQ_10_ is commercially produced through chemical synthesis, but its production using microorganisms is potentially a less complex alternative. The production of CoQ_10_ using certain purple non-sulfur bacteria has been reviewed [9] and the entire biosynthetic pathway for its production has been elucidated in a metabolically engineered bacterium, *Corynebacterium glutamicum* [10].

Different metabolic pathways are involved in the biosynthesis of the polyisoprenoid chain and polar ring of CoQ, and the modification of the polar ring after it has attached to the hydrophobic chain. In *E. coli*, the *p*-hydroxybenzoate (*p*HB) precursor of CoQ is synthesized through the action of chorismate–pyruvate–lyase (EC 4.1.3.40) on chorismite, a product of the shikimate pathway [11]. In higher eukaryotes, *p*HB is synthesized from tyrosine or phenylalanine [12,13], whereas in *S. cerevisiae*, *p*-aminobenzoate (*p*ABA) is an alternate precursor [14]. Precursors of the isoprenoid chain are isopentenyl diphosphate (IPP) and its isomer dimethylallyl diphosphate (DMAPP). Both these are made in the mevalonate pathway [15,16], or the methylerythritol 4-phosphate pathway in prokaryotes [17], depending on the species. The attachment of the polyisoprenoid chain to the C-3 position of *p*HB or *p*ABA is catalyzed by intramembrane prenyltransferases [18]. The ring is further hydroxylated, methylated, and decarboxylated. In eukaryotes, the enzymes involved in the modification of the polar ring occur together in the CoQ synthome, a multi-enzyme complex [19].

Certain bacteria (*Agrobacterium tumefaciens* [20]; *Rhodobacter sphaeroides* [21]; *Paracoccus denitrificans* [22]; *Sphingomonas* sp. [23]; *Proteus* spp. [24]) and some yeasts (*Candida* spp. [25]; *Rhodotorula* spp. [26]) are known to produce CoQ_10_. Among microorganisms, the highest CoQ_10_ content (11.8 mg g^−1^ dry biomass) has been reported in *A. tumefaciens* [27]. This bacterium is potentially attractive for producing CoQ_10_ because it can be grown to a relatively high biomass concentration, exceeding 50 g L^−1^ [28].

Thraustochytrids are unicellular heterotrophic microorganisms that degrade organic matter. Certain marine thraustochytrids are used for the commercial production of the polyunsaturated fatty acid docosahexaenoic acid (DHA) [29]. Under suitable culture conditions, the lipid content of thraustochytrids may exceed 55% of the dry cell mass [29,30,31]. In fed-batch culture on sugars, the thraustochytrid biomass can be grown to a dry mass concentration exceeding 50 g L^−1^ [29]. In addition to DHA, thraustochytrids produce various other compounds of potential commercial interest (e.g., squalene, β-carotene, astaxanthin, exopolysaccharides) [32,33]. This work characterized the production of CoQ_10_ by the thraustochytrid *Thraustochytrium* sp. RT2316-16 [34]. The production of CoQ_10_ has not been reported in any thraustochytrid.

## 2. Results

### 2.1. Identification and Quantification of CoQ_10_

For the identification of CoQ_10_ in the biomass, the extracted lipid fraction was first subjected to thin-layer chromatography. The lipids that migrated with the solvent front contained CoQ_10_ based on the HPLC analysis. The presence of CoQ_10_ in this fraction was further confirmed via UPHLC-MS/MS. The full-scan mass spectra showed peaks at *m/z* values of 866.18 and 860.84, consistent with the molar masses of ubiquinol (the reduced form of ubiquinone Q_10_) at 865.36 g gmol^−1^, and ubiquinone Q_10_, 863.34 g gmol^−1^.

The HPLC analysis was used for the identification and quantification of CoQ_10_. The chromatograms of the lipids extracted from RT2316-16 had peaks consistent with the measured retention times of the authentic CoQ_10_ and CoQ_9_. 

### 2.2. Effects of the Nitrogen Source on the CoQ Content in RT2316-16

**Figure 1:**
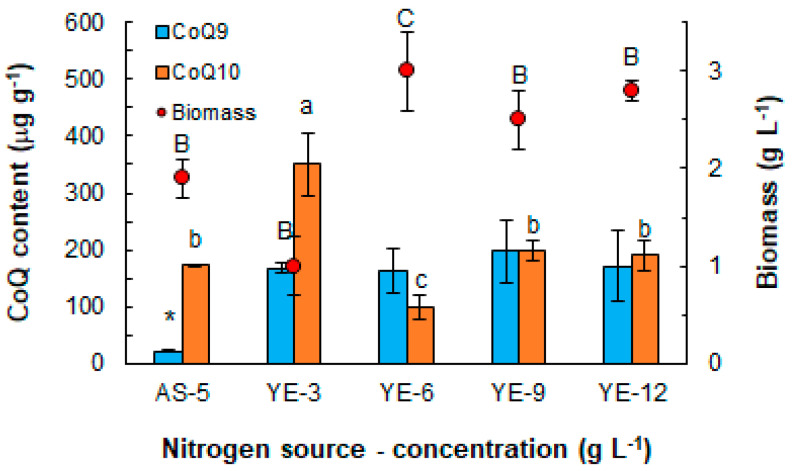
Effect of the nitrogen source (yeast extract, YE, or ammonium sulphate, AS), and yeast extract concentration (3, 6, 9 and 12 g L^−1^) on the CoQ (CoQ_9_ and CoQ_10_) in the biomass of RT2316-16. The biomass was grown for 36 h in media comprising glucose (20 g L^−1^) in diluted (50% *v v*^−1^) artificial seawater (the other components except for yeast extract were as noted in Section 4.1). All media contained monosodium glutamate (MSG) at 0.6 g L^−1^. A different letter above a bar or symbol (*) indicates that the value in the series was significantly (*p* < 0.05) different.

The effects of the nitrogen source (either yeast extract (6 g L^−1^ as in the control medium) or ammonium sulfate (5 g L^−1^, as used in an earlier work [35])) on the CoQ content of the biomass were assessed in a medium that contained glucose (20 g L^−1^). After 36 h of incubation, the biomass concentration (3.0 ± 0.4 g L^−1^) in the yeast extract containing the medium was 37% higher than the concentration in the medium with ammonium sulfate (Figure 1).

The effect of the nitrogen source on the CoQ_9_ and CoQ_10_ in the biomass was different (Figure 1): the CoQ_9_ content was higher (163 ± 39 µg g^−1^) in the biomass grown with the yeast extract (6 g L^−1^, the control medium), whereas the biomass grown with ammonium sulfate had a higher CoQ_10_ content (174 ± 1 µg g^−1^).

Because a higher biomass concentration could be obtained using the yeast extract, a necessary requirement for generating a high volumetric concentration of an intracellular product, the effect of the yeast concentration was further evaluated (Figure 1). After the 36 h of incubation, the differences in the biomass concentration were not significant (*p* > 0.05) (average biomass concentration 2.8 g L^−1^) if the concentration of the yeast extract exceeded 3.0 g L^−1^ (Figure 1). The lowest concentration of the yeast extract (i.e., 3.0 g L^−1^) that still allowed for the biomass to grow at a slow pace resulted in a biomass with the highest CoQ_10_ content (350 ± 1 µg g^−1^). On the other hand, for the yeast extract concentrations exceeding 3.0 g L^−1^, the concentration had no significant effect (*p* > 0.05) on CoQ_9_ in the biomass and the average content of CoQ_9_ was 175 µg g^−1^ (Figure 1).

### 2.3. Precursors of CoQ Ring Were Not Required for Its Biosynthesis in RT2316-16

In some bacteria, *p*-hydroxybenzoic acid (*p*HB) is a well-known precursor of the quinone ring in CoQ. Therefore, the effect of providing *p*HB in the culture medium, on the production of CoQ was evaluated in media containing the yeast extract (the control medium), or ammonium sulfate. All other media components were provided at their normal concentrations (Section 4.1). At a *p*HB concentration of 25 mg L^−1^, a value that was 30% less than the reported optimum concentration for producing the coenzyme Q_8_ in *E. coli* [36], the growth of RT2316-16 was inhibited in both media. Thus, *p*HB was added 72 h after the inoculation to circumvent the inhibitory effect. In the medium with the yeast extract, 24 h after the *p*HB was added, CoQ_10_ in the biomass increased to 158 ± 19 µg g^−1^ (from nil, just before the *p*HB was added), whereas CoQ_9_ in the biomass decreased to 3 µg g^−1^ (from around 22 µg g^−1^) (Table 1).

Under otherwise equivalent conditions, CoQ_10_ in the biomass decreased from 800 ± 12 µg g^−1^ (at 72 h, before *p*HB was added) to 151 ± 3 µg g^−1^ (at 96 h) in the medium with ammonium sulfate (Table 1). 

Some yeasts are known to use *p*-aminobenzoic acid (*p*ABA) as a precursor for the quinone ring in CoQ [14]. Because *p*ABA was a component of one of the vitamin solutions (solution I; Section 4.1) normally used for growing the thraustochytrid RT2316-16, its effect was evaluated using a *p*ABA-free medium that was otherwise identical to the control medium. The *p*ABA-free medium reduced the growth rate of RT2316-16 relative to the control medium, and at 36 h, the biomass concentration was 39% lower than the concentration obtained with the control medium (Table 1). Although, *p*ABA significantly affected the biomass concentration, the CoQ_9_ content (at 36 h) in the biomass was not significantly (*p* > 0.05) affected if *p*ABA was excluded (the average CoQ_9_ content was 169 µg g^−1^). In contrast, at 36 h, the CoQ_10_ in the biomass grown without *p*ABA was 4.9-fold higher (489 ± 55 µg g^−1^) than in the biomass grown with *p*ABA (Table 1).

### 2.4. The CoQ Content in the Biomass: Influence of the Growth Stage and the Composition of the Culture Medium

**Figure 2:**
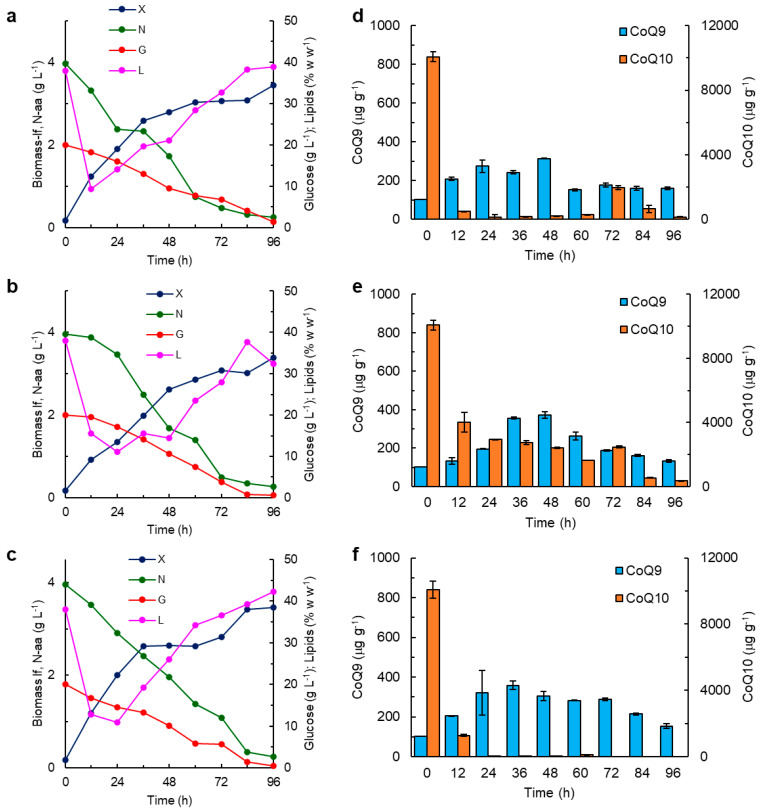
Time profiles of the concentrations of the lipid-free biomass (X), residual glucose (G), residual amino acids (N) and the total lipid content of the biomass (L) of *Thraustochytrium* RT2316-16 (**a**–**c**), and the content of CoQ_9_ and CoQ_10_ in the biomass (**d**–**f**). The media formulations were as follows: (**a**,**d**) control medium supplemented with vitamin solution II that contains *p*ABA; (**b**,**e**) control medium supplemented with vitamin solution II without *p*ABA; (**c**,**f**) control medium supplemented with vitamin solution II without *p*ABA, but with folic acid (50 μg L^−1^).

A set of three growth experiments was conducted to elucidate the observed positive effect of *p*ABA on the growth of RT2316-16, and the simultaneous negative effect on the CoQ_10_ content of the biomass (results in Table 1). Thraustochytrid was grown in media that differed only due to the supplied vitamins: (1) all vitamins provided, including *p*ABA; (2) all the same vitamins as in 1, but without *p*ABA; and (3) the same vitamins as in 2, but including folic acid (50 µg L^−1^). The changes in the vitamin composition in the media affected the growth of the lipid-free biomass (Figure 2a–c).

During the early part of the culture, the concentration of the lipid-free biomass in the medium with *p*ABA increased at a specific growth rate (0.111 h^−1^, calculated as the slope of the lipid-free biomass concentration natural logarithm curve versus time) (Figure 2a) that exceeded the specific growth rate observed in the *p*ABA-free medium (0.074 h^−1^) (Figure 2b). In the medium with the folic acid (Figure 2c), the concentration of the lipid-free biomass increased at a specific growth rate comparable to that in the *p*ABA-containing medium until 36 h, and afterward the biomass concentration remained nearly constant (2.6 ± 0.0 g L^−1^) until 60 h, and then the growth resumed. Despite these differences, the final concentration of the lipid-free biomass in the three media was not significantly (*p* > 0.05) different, and the average concentration was 3.4 ± 0.1 g L^−1^.

A substantial reduction in the lipid content in the biomass was observed during the first 12 h of incubation (Figure 2a–c). This period of lipid loss in the *p*ABA-free medium with the folic acid extended to 24 h of incubation. After 96 h, the biomass of RT2316-16 grown with folic acid reached the highest lipid content (43.3 ± 1.4%) (Figure 2c); this lipid content was 10% more than the lipid content of the biomass grown with *p*ABA (Figure 2a). During 12 to 48 h of incubation, the lipid content in the biomass grown without the *p*ABA did not increase, and the average lipid content during this period was 14.1% (Figure 2b); nevertheless, after 84 h, the total lipid content reached 37.6 ± 0.9%, decreasing to 32.5 ± 0.8% after 96 h (Figure 2b).

The CoQ_9_ content in the biomass varied with the incubation time: there was a peaking pattern in the CoQ_9_ content, although the peaks occurred at different times in the different media (Figure 2d–f). The highest CoQ_9_ content in the biomass occurred as follows: 314 ± 2 µg g^−1^ at 48 h in the biomass grown with *p*ABA (Figure 2d); 359 ± 23 µg g^−1^ at 36 h in the biomass grown with folic acid (Figure 2f); and 371 ± 18 µg g^−1^ at 48 h in the biomass grown without *p*ABA (Figure 2e). The biomass used for the inoculation (i.e., at 0 h) was high in CoQ_10_ (10,075 ± 87 µg g^−1^). After 12 h, the CoQ_10_ content decreased to 492 µg g^−1^ in the biomass grown with *p*ABA (Figure 2d) and remained at less than 296 µg g^−1^ until 60 h of incubation. CoQ_10_ in the biomass grown without *p*ABA remained at >2400 µg g^−1^ until 72 h of incubation (Figure 2e), whereas the biomass grown with folic acid had the lowest CoQ_10_ content (the average was 40 µg g^−1^), and no CoQ_10_ was detected after 60 h (Figure 2f).

**Figure 3:**
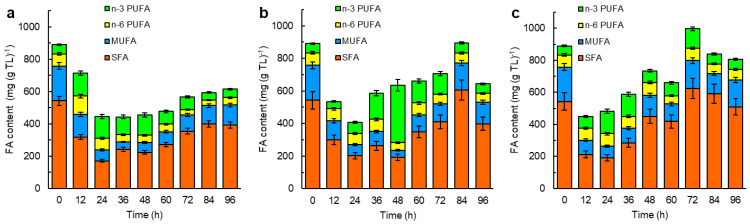
Time profiles of the fatty acid (FA) composition of the total lipids (TLs) of the biomass of RT2316-16 grown in the control medium supplemented with: (**a**) vitamin solution II that contained *p*ABA; (**b**) vitamin solution II without *p*ABA; and (**c**) vitamin solution II without *p*ABA but with folic acid (final concentration = 50 μg L^−1^). Abbreviations: SFA, saturated fatty acids; MUFA, monounsaturated fatty acids; PUFA, polyunsaturated fatty acids; n-3, omega-3 (eicosapentaenoic acid (EPA) + docosapentaenoic acid (DPA) + docosahexaenoic acid (DHA)); n-6, omega-6 (linoleic acid + γ-linolenic acid + cis-8,11,14-eicosatrienoic acid + arachidonic acid).

The fatty acid composition of the total lipids in the biomass grown in the media with the different vitamins changed with incubation time (Figure 3).

In all three cases, the fraction of saturated fatty acids and monounsaturated fatty acids in the total lipids decreased during the first 24 h. Afterward, both fatty acid fractions began to increase, notwithstanding the differences in the culture media (Figure 3). At 72 h, the fatty acid content in the total lipids was the lowest (567 ± 25 µg g^−1^) in the total lipids recovered from the biomass grown with *p*ABA (Figure 3a). The total fatty acids in the total lipids were the highest (990 ± 31 µg g^−1^) in the 72 h biomass grown with folic acid (Figure 3c). The total lipids with the highest fraction of the long-chain omega-3 polyunsaturated fatty acids (351 ± 12 µg g^−1^) were those recovered at 48 h from the biomass grown without *p*ABA (Figure 3b). This was the biomass from the phase of the arrested growth (24–60 h) of the lipid-free biomass (Figure 2b).

**Figure 4:**
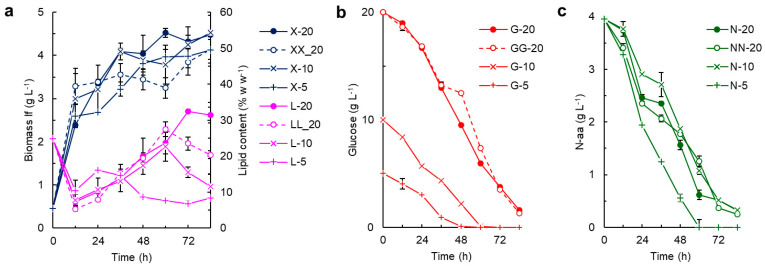
Effect of the initial concentration of glucose (20, 10 and 5 g L^−1^) on (**a**) the concentration of the lipid-free biomass (X) and the total lipid (L) in the biomass; (**b**) the residual glucose concentration (G); and (**c**) the residual concentration of amino acid nitrogen (N-aa). The X, L, G and N data obtained in media without the vitamin solution I and with an initial glucose concentration of 20 g L^−1^ are denoted as XX-20, LL-20, GG-20 and NN-20, respectively.

In a further set of experiments, the effects of the initial glucose concentration (5, 10 and 20 g L^−1^) on the growth, nutrient consumption and the CoQ content of the RT2316-16 biomass were assessed in the media of the same composition as the control medium (Figure 4).

Effects of omitting vitamin solution I (containing thiamine (vitamin B1), Ca-pantothenate (vitamin B5), nicotinic acid (vitamin B3) and pyridoxine (vitamin B6)) from the culture medium were assessed using a medium with an initial glucose concentration of 20 g L^−1^ and the other components at the level specified for the control medium. The initial concentration of glucose had a major effect on the total lipid in the biomass: whenever the glucose concentration decreased to a certain value (apparently dependent on the initial concentration of glucose) (Figure 4b), the total lipid in the biomass began to decrease (Figure 4a). Although the final concentration of the lipid-free biomass was lower when the initial concentration of glucose reduced in the above-specified range (Figure 4a), the differences were not significant (*p* > 0.05) and the average final concentration of the lipid-free biomass was 4.4 ± 0.4 g L^−1^. A similar final concentration of the lipid-free biomass (4.3 ± 0.5 g L^−1^) was found in the medium free of vitamin solution I (Figure 4a). This slight effect of glucose concentration on the concentration of the lipid-free biomass could be explained as follows: all media had the same initial concentration of the yeast extract, the nitrogen source; however, the rate of consumption of the nitrogen source by RT2316-16 (Figure 4c) was affected by the initial concentration of glucose (Figure 4b), and the lowest initial glucose concentration (5 g L^−1^) resulted in a rapid and complete consumption of nitrogen after 60 h (Figure 4c). Omission of the above-specified B vitamins (vitamin solution I) from the culture medium promoted a rapid growth of the lipid-free biomass that attained a concentration of 3.3 ± 0.4 g L^−1^ in 12 h (Figure 4a). After this period of rapid growth, the concentration of the lipid-free biomass remained nearly constant until 60 h (Figure 4a), but the total lipids in the biomass increased from 5.3 to 27.5% (Figure 4a), implying that glucose was being consumed (Figure 4b) to support lipid production rather than growth. The arrest in the growth of the lipid-free biomass after 12 h may have prompted a change in the cell metabolism, resulting in a second phase of growth after 60 h supported by the yeast extract, a source of both nitrogen and carbon (Figure 4c). During this phase of growth, the total lipid content of the biomass declined from 27.5 to 20.4% (Figure 4a). 

**Figure 5:**
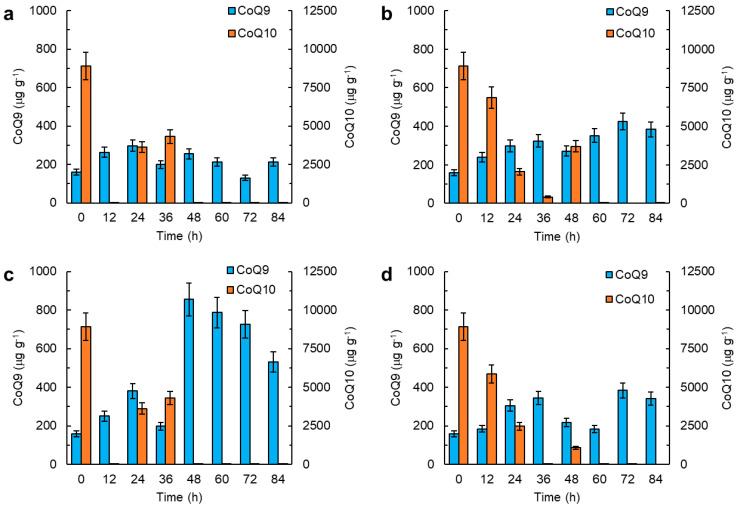
CoQ_9_ and COQ_10_ in the biomass of RT2316-16 at various times during culturing. The biomass was grown in media (with vitamin solution I) with the following initial concentrations of glucose (g L^−1^): (**a**) 20; (**b**) 10; and (**c**) 5. (**d**) The biomass was grown with a glucose concentration of 20 g L^−1^ without vitamin solution I.

The biomass specific amounts of CoQ_9_ and CoQ_10_ varied during growth in a manner that depended on the composition of the medium (Figure 5a–c).

Overall, CoQ_9_ in the biomass grown with a low initial concentration of glucose (Figure 5c) nearly doubled after the glucose was exhausted (Figure 4b). Omission of the B vitamins (i.e., vitamin solution I) promoted an increase in the CoQ_9_ content of the biomass, particularly during the last 24 h of culture (Figure 5d). In these experiments, CoQ_10_ in the biomass just after inoculation was 8900 µg g^−1^, but no CoQ_10_ was detected after 60 h (Figure 5a–d). An initial glucose concentration of 10 g L^−1^ resulted in a steady decrease in CoQ_10_ in the biomass until 36 h (Figure 5b). A similar pattern was observed in the vitamin-deficient media with an initial glucose concentration of 20 g L^−1^ (Figure 5d).

## 3. Discussion

Coenzyme Q (CoQ) is a water insoluble molecule composed of a redox-active benzoquinone ring attached to a polyisoprenoid chain. Intake of CoQ_10_ may benefit patients undergoing the treatment of certain diseases; however, CoQ_10_ is not approved by the United States Food and Drug Administration for the treatment of any medical condition [37]. This notwithstanding, CoQ_10_ is widely available as a nutritional supplement [38]. Alternatives to the current chemical synthesis are needed for producing CoQ_10_, and microorganisms provide some potentially useful production options [9,10]. 

CoQ biosynthesis involves multiple reactions, including biosynthesis of the polyisoprenoid chain and *p*-hydroxybenzoate (*p*HB). In addition, ring modifications occur after the linking of the polyisoprenoid chain and 6-carbon ring in a highly regulated process. The present study showed that the thraustochytrid *Thraustochytrium* sp. RT2316-16 produced two CoQs, i.e., CoQ_9_ and CoQ_10_, differing in the length of the polyisoprenoid chain. The occurrence of two CoQs in an organism is not uncommon and has been reported in plants such as *Salvia miltiorrhiza* [39]. In humans, CoQ_10_ is the dominant form, but 2–5% of total CoQ occurs as CoQ_9_ [40]. The synthesis of the polyisoprenoid chain is catalyzed by trans-polyprenyl diphosphate synthases that use an allylic diphosphate as a primer molecule for the consecutive addition of isopentenyl diphosphate molecules until the required chain length is achieved. The linking of the polyisoprenoid chain to 4-hydroxybenzoate (*p*HB) is catalyzed by 4-hydroxybenzoate (*p*HB)-polyprenyl diphosphate transferases (EC 2.5.1.39). The length of the polyisoprenoid chain in CoQ is defined by trans-polyprenyl diphosphate synthases rather than by the enzyme that catalyzes the linking of *p*HB and the polyprenyl diphosphate chain [41]. The size of the active site in trans-polyprenyl diphosphate synthases is likely the main factor determining the chain length [42].

In RT2316-16, CoQ_9_ was detected in the biomass grown in all the culture media (Figure 1, Figure 2 and Figure 5; Table 1). In an earlier study, only CoQ_9_ was found in *Thraustochytrium* sp. ONC-T18 [43]. CoQ_10_ was detected in the biomass recovered from some of the media and, in some cases, at certain times during incubation. The CoQ_10_ content in RT2316-16 varied dramatically with the incubation time (Figure 2 and Figure 5). The differences in occurrences of the two CoQs and the time-dependent changes in their concentrations in the biomass were probably associated with their different functions, possibly in relation to the membranes of the specific organelles (e.g., Golgi body, lysosome, and peroxisome) harboring them. CoQ is involved in the control of reactive oxygen species in diverse processes, including during the formation of the disulfide bonds (DsbA-DsbB oxidation-reduction pathway); disulfide oxidation (sulfide–quinone reductase); pyrimidine metabolism (dihydroorotate dehydrogenase) [44]; and the metabolism of branched amino acids [45]. CoQ participates in ATP production under conditions that favor the β-oxidation of fatty acids (electron-transferring flavoprotein–ubiquinone oxidoreductase). The noticeable reduction in the total lipid content in the biomass of RT2316-16 observed during the first hours after inoculation (Figure 2 and Figure 4) may have been due to the oxidation of fatty acids.

The relative proportion of the two CoQs in the biomass varied with the culture medium and incubation time (Table 1; Figure 1, Figure 2 and Figure 5). In media with the same initial concentration of glucose (20 g L^−1^), a low initial concentration of the yeast extract (3 g L^−1^) produced a biomass with high CoQ_10_ content. In contrast with this, no significant effect of the initial yeast extract concentration was observed on the CoQ_9_ content of the biomass, irrespective of the yeast extract concentration used (3, 6, 9 or 12 g L^−1^) (Figure 1). The different effects of the yeast extract concentration on the two CoQs suggest that their synthesis was regulated by different signals.

In studies with certain bacteria (*Agrobacterium tumefaciens* [46], *Azotobacter vinelandii* [47], and *Rhodospirillum rubrum* [48]), ammonium sulfate alone, or in combination with yeast extract, stimulated the production of CoQ_10_. In the present work utilizing RT2316-16, ammonium sulfate also enhanced CoQ_10_ in the biomass (Figure 1), but ammonium sulfate tended to suppress the growth of RT2316-16 and was therefore not further investigated. 

The best-known precursor for the benzoquinone ring in CoQ is *p*HB [49]; however, its use is limited by its growth inhibitory effect both on RT2316-16 and other diverse microorganisms. The *p*HB supplementation (25 mg L^−1^) of the culture during the late stage of growth (72 h of incubation) enhanced the CoQ_10_ content of the yeast *Sporidiobolus johnsonii* [50]. In the present work utilizing RT2316-16, the *p*HB feeding had different effects on CoQ_9_ and CoQ_10_ in the biomass depending on the culture medium (Table 1). Supplementation with the precursor after 72 h of growth did not affect the production of CoQs if the culture media contained the yeast extract (Table 1). The lack of biomass growth after *p*HB was added in the medium with ammonium sulfate 72 h after inoculation (Table 1) was a possible explanation for the arrested CoQ synthesis that, combined with a relatively short half-life of CoQ, resulted in its decrease in the cell (Table 1). 

The additive with the most significant effects on the CoQ_10_ content of RT2316-16 was *p*ABA, a compound that is commonly included among the vitamins to promote the growth of thraustochytrids. In the present study, the biomass supplied with *p*ABA had less CoQ_10_ compared with the biomass grown in *p*ABA-free media (Figure 2). As *p*ABA had no significant effect on CoQ_9_ in the biomass of RT2316-16, it could not have been a precursor of the CoQ ring, unlike what has been reported in the yeasts *S. cerevisiae* and *Schizosaccharomyces pombe* [14,51]. On the other hand, the CoQ_9_ content of RT2316-16 grown in media with and without *p*ABA did not differ significantly (Figure 2); therefore, the *p*ABA inhibition of 4-hydroxybenzoate (*p*HB)-polyprenyl diphosphate transferases involved in the synthesis of CoQ_9_ could be ruled out, although such inhibition has been observed in mammalian cells [52]. Although different enzymes may be involved in the synthesis of CoQ_9_ and CoQ_10_, most polyprenyl diphosphate transferases accept substrates with a range of polyisoprenoid chain lengths, but are highly specific for *p*HB [53,54,55].

The results showed that *p*ABA, or folic acid (without *p*ABA), had a positive effect on biomass growth and the total lipid content of the RT2316-16 biomass (Figure 2 and Figure 4). In plants, *p*ABA, 6-hydroxymethyl dihydropterin and glutamate are precursors of folate, a generic term that encompasses tetrahydrofolate (THF), the most reduced form, and its derivatives. Unlike folate, folic acid (the acid form of folate) has no biological activity; to become active, folic acid is reduced by dihydrofolate reductase (EC 1.5.1.3). Folates act as acceptors and donors of one-carbon units in multiple reactions, including the synthesis of nucleic acids (thymidine and purines), amino acids, pantothenate, and S-adenosylmethionine (SAM). SAM-dependent methylation reactions are required for post-translational modifications of proteins, DNA methylation (modification of gene expression), and synthesis of hormones, creatine, carnitine, and phosphatidylcholine. In thraustochytrids phosphatidylcholine is involved in the assembly of long-chain polyunsaturated fatty acids into glycerolipids [56]. The results showed that *p*ABA and folic acid affected the profile of fatty acids in RT2316-16 (Figure 3).

Folic acid eliminated the effects of omitting *p*ABA from the culture medium, increasing the growth rate of the lipid-free biomass and accumulation of total lipids in the biomass (Figure 2). The multiple roles of folates in the biosynthesis of nucleic acid and SAM could explain the higher growth rate of lipid-free biomass in cultures supplemented with *p*ABA or folic acid. The relationship between the availability of *p*ABA or folic acid and total lipid synthesis was more difficult to explain. In the presence of *p*ABA and folic acid, more of the carbon consumed was channeled to lipid synthesis through mechanisms that were not elucidated.

Although *p*ABA and folic acid had a significant effect on the specific growth rate in the early hours of incubation (Figure 2), the CoQ_10_ content of the biomass from these early stages (Figure 2d,f) was quite low in comparison with CoQ_10_ in the biomass grown without *p*ABA (Figure 2e). This pattern suggested an imbalance between the rates of synthesis and degradation of CoQ_10_. For example, in plant cells, the half-life of CoQ is around 30 h [57] and therefore the cells must synthesize this electron transporter continuously. Another possibility is that CoQ_10_ was not required under the conditions that promoted the rapid growth of the biomass.

The initial concentration of glucose had a significant effect on the lipid content of the biomass (Figure 4a). As soon as the glucose concentration declined to less than 5 g L^−1^, the mobilization of the lipids began, so long as the nitrogen supply had not been exhausted (Figure 4). The lowest glucose concentration (5 g L^−1^) promoted a rapid consumption of the nitrogen source and a subsequent increase in CoQ_9_ in the biomass upon exhaustion of the nitrogen supply (Figure 5c). The CoQ_10_ content was the highest in the biomass at inoculation, but decreased during incubation to less than 20 μg g^−1^ by 12 h, only if the initial concentration of glucose was either 20 g L^−1^ or 5 g L^−1^ (Figure 5a,c). If the initial glucose concentration was 10 g L^−1^, with the full vitamins, and 20 g L^−1^ without some of the vitamins, the decrease in the CoQ_10_ content of the biomass during the first 12 h of incubation was low (Figure 5b,d). This may have been due to the ceased growth after 12 h (Figure 4a). This pattern suggests that the decrease in the CoQ_10_ content of the biomass was promoted by growth. Okada et al. [58] showed that CoQ played an essential role in embryo development in plants (*Arabidopsis*), and this effect was also observed in mammals. 

In view of the results presented here, the thraustochytrid RT2316-16 is a potential vehicle for producing CoQ_10_. A CoQ_10_ content close to 10 mg g^−1^ was achieved in the biomass under non-optimized culture conditions. In comparison with this, a CoQ_10_ content of around 8.7 mg g^−1^ was obtained in the bacterium *Rhodobacter sphaeroides* KY-4113 under optimized culture conditions [59]. Similarly, in *Agrobacterium tumefaciens* KCCM 10,413, the CoQ_10_ content could be maximized to nearly 11.8 mg g^−1^ [60]. In principle, there is the potential to further enhance the production of CoQ_10_ in RT2316-16. This can be achieved either through an empirical optimization approach involving a multivariate statistical optimization of the culture conditions or a more rational optimization of production might be possible by identifying the roles of the two CoQs in RT2316-16 and by characterizing the factors affecting the activity of the prenyltransferases involved in the synthesis of the isoprenoid chains of CoQs. 

A relationship has been suggested to exist between the length of the hydrophobic polyisoprenoid chain of CoQ and the hydrophobicity of the membrane of the organism synthesizing CoQ [58]. Furthermore, the difference between the crystallization temperatures of CoQ_9_ (0.5 °C) and CoQ_10_ (9.7 °C) might explain the preponderance of CoQ_9_ in the organisms adapted to cold environments [61]. In this context, it is worth noting that the thraustochytrid RT2316-16 was isolated from the cold Antarctic environment, although how the temperature might affect the CoQ content of its biomass is yet to be assessed.

## 4. Materials and Methods

### 4.1. Microorganism and Inoculum Preparation

*Thraustochytrium* sp. RT2316-16 was used in all experiments. The isolation and identification of this marine thraustochytrid were previously reported [34,62]. The pure stock cultures were kept frozen at −80 °C in 50% (*v v*^−1^) aqueous glycerol.

The inoculum for the experiments was grown aseptically in Erlenmeyer flasks (250 mL) containing 100 mL of a sterile medium of the following composition (g per liter of a 1:1 (*v v*^−1^) mixture of distilled water and artificial seawater, ASW): glucose (Merck, Darmstadt, Germany) 20, yeast extract (Merck) 6, and monosodium glutamate (Merck) 0.6. ASW contained the following per liter of distilled water: NaCl 27.5 g, MgCl_2_·6H_2_O 5.38 g, MgSO_4_·7H_2_O 6.78 g, KCl 0.72 g, NaHCO_3_ 0.2 g and CaCl_2_·2H_2_O 1.4 g. A trace elements solution (24 mL L^−1^), and two vitamin solutions (vitamin solution I, 3.6 mL L^−1^; vitamin solution II, 3.6 mL L^−1^), were filter-sterilized by passing through a 0.2 μm sterile membrane filter and added to the medium. The trace element solution contained the following in distilled water (g L^−1^): MnCl_2_·4H_2_O 0.3, ZnSO_4_·7H_2_O 0.3, CoCl_2_·6H_2_O 0.004, CuSO_4_·5H_2_O 0.2, NiSO_4_·6H_2_O 0.2, FeSO_4_·7H_2_O 1 and KH_2_PO_4_ 5 [63]. Vitamin solution I contained the following in distilled water (g L^−1^): thiamine 0.04, Ca-pantothenate 0.02, nicotinic acid 0.02 and pyridoxine 0.008 (Sigma-Aldrich, St Louis, MO, USA) [63]. Vitamin solution II contained the following in distilled water (g L^−1^): biotin 0.01, cobalamin (vitamin B12) 0.001, riboflavin 0.1, pyridoxamine 0.2 and *p*-aminobenzoic acid 0.02 (Sigma-Aldrich) [63]. Of a thawed stock culture, 1 mL was added to 100 mL of the above medium and the flask was incubated (15 °C) aseptically on an orbital shaker (150 rpm) for 4 days. All experiments used the inocula prepared as explained above. The medium used for the inoculum was denoted as the control medium. All experiments used this medium, or one of its modified forms. 

### 4.2. Effect of the Culture Medium Composition on the Production of CoQ_10_

In the first set of experiments, RT2316-16 was cultured in the control medium, the control medium with the yeast extract replaced by ammonium sulfate (5 g L^−1^) (the ammonium sulfate medium) and the control medium with higher concentrations of yeast extract (3, 9 and 12 g L^−1^). Cultures were grown in triplicate in 250 mL Erlenmeyer flasks, each containing 100 mL of the sterile medium and an identical inoculum. Incubation conditions were as specified above for the inoculum. After 36 h, the culture from each flask was centrifuged (6000× *g*, 4 °C, 10 min), the recovered biomass was washed with distilled water, freeze-dried, weighed and stored at −20 °C for lipid analysis.

In the second set of experiments, the control medium was used to test the effects of a late supplementation with *p*HB at a final concentration of 25 mg L^−1^. A culture grown using the ammonium sulfate medium was also used to test the same effect. All cultures were grown in triplicate in 250 mL Erlenmeyer flasks, each containing 100 mL of the sterile medium and an identical inoculum. Incubation conditions were the same as for the inoculum. After 72 h of incubation, 2 culture flasks were harvested (centrifugation at 6000× *g*, 4 °C, 10 min): one flask had the culture grown in the control medium and one flask the culture grown in the ammonium sulfate medium. The two remaining flasks of the cultures grown in the control medium and the two flasks of the culture grown in the ammonium sulfate medium were each supplemented with *p*HB at a final concentration of 25 mg L^−1^, and incubation was continued for a further 24 h. All cultures were then harvested for the biomass. The recovered biomass was washed and stored for analysis, as specified earlier. 

The effect of *p*ABA and folic acid on growth were tested. For this, Erlenmeyer flasks (250 mL total volume, 100 mL medium) were inoculated using identical inocula to measure the growth curves. The following media were used: 16 flasks with the control medium made by omitting *p*ABA in vitamin solution II; and 16 flasks with the control medium made by omitting *p*ABA in vitamin solution II, but with folic acid added at a concentration of 50 μg L^−1^ in the medium. For comparison, 16 flasks, each with 100 mL of the control medium, were run in parallel. Incubation conditions were as specified earlier for the inoculum. Two flasks of each treatment were sacrificed each for 12 h to recover the biomass for lipid analysis. In addition, 10 mL of the cell-free culture supernatant was stored (−20 °C) for the analysis of residual glucose and residual amino acid nitrogen.

To test the effect of the initial glucose concentration on biomass growth, Erlenmeyer flasks (250 mL total volume, 100 mL medium) were inoculated (identical inocula). The flasks all had the control medium, but with different initial concentrations of glucose: 14 flasks with initial glucose = 5 g L^−1^; 14 flasks with initial glucose = 10 g L^−1^; and 14 flasks with initial glucose = 20 g L^−1^. Fourteen other flasks (250 mL total volume, 100 mL medium) with identical inocula were run in parallel using initial glucose concentrations = 20 g L^−1^, but without vitamin solution I. Two flasks of each treatment were sacrificed each for 12 h to recover the biomass for lipid analysis. A sample (10 mL) of the cell-free culture supernatant from each flask was stored (−20 °C) for the measurements of residual glucose and amino acid nitrogen. 

### 4.3. Analyses

#### 4.3.1. Cell Dry Weight

The concentration of biomass was determined gravimetrically. For this, a known culture volume (2 mL) was centrifuged (2057× *g*, 10 min), the cell pellet was washed twice with distilled water, recovered via centrifugation and dried to constant weight at 105 °C.

#### 4.3.2. Residual Reducing Sugars

The residual concentration of glucose in the cell-free medium was measured via HPLC (Alliance Waters e2695 separation module, Waters, Mildford, MA, USA) analysis using a SHODEX KS 800 column (Showa Denko KK, Tokyo, Japan) maintained at 80 °C. The mobile phase was deionized water at a flow rate of 1 mL min^−1^. A refractive index detector (model 2414; Waters, Mildford, MA, USA) was used. The identification and quantification were carried out by comparing the retention times and the areas under the peaks with those of standard solutions.

#### 4.3.3. Residual Amino Acids

The residual concentrations of amino acids were determined using the *o*-phthaldialdehyde method [64]. The reaction solution was prepared with *o*-phthaladehyde (OPA, Sigma), 2-mercaptoethanol and 50 mM carbonate buffer. Of the reaction solution, 1 mL was mixed with 100 µL of the sample, incubated at 25 °C for 1 to 2 min, and the spectrophotometric absorbance was measured at 340 nm. A calibration curve was made using standard solutions of the yeast extract in the concentration range of 0 to 1 g L^−1^.

#### 4.3.4. Extraction and Quantification of CoQ

A 50 mg sample of the freeze-dried powdered biomass was suspended in 6 mL of cold methanol (−20 °C) and sonicated (20 min). Petroleum ether (6 mL) was added, the suspension was vortex-mixed (1 min) and centrifuged (4000× *g*, 4 °C, 10 min). The upper phase was withdrawn and a second extraction with petroleum ether (3 mL) was made; the extracts were pooled in a vial. The solvent in the pooled extract was evaporated under a stream of nitrogen. The residual lipids were suspended in 450 µL of a solvent mixture (methanol: ethanol, 65:35 *v v*^−1^), and 50 µL of a ferric chloride (FeCl_3_) solution (1% *w v*^−1^ in ethanol) was added. The sample was filtered with a 0.22 µm syringe filter before HPLC analysis.

The HPLC analysis employed a published method [65]. A C18 HPLC column (250 × 4.6 mm, 5 µm; Symmetry C18, Waters, Milford, MA, USA) and an ultraviolet detector (275 nm) (Waters 2487 dual detector) were used. The mobile phase comprised a mixture of methanol: ethanol (65:35 *v v*^−1^) at a flow rate of 1.0 mL min^−1^. The column temperature was 30 °C and the injection volume was 20 µL. Standard solutions of CoQ_10_ and CoQ_9_ (Sigma-Aldrich) were used for the identification and quantification.

#### 4.3.5. UHPLC-MS/MS Analysis

The total lipids were dissolved in chloroform at a concentration of 10 mg mL^−1^. An aluminum thin-layer chromatography (TLC) plate (silica gel 60 F254; Merck) was washed with the mobile phase (petroleum ether: chloroform 20:80 *v v*^−1^) and dried at room temperature. A 20 μL portion of the lipid solution was loaded on the plate and eluted with the above-specified mobile phase. The TLC plate was developed under iodine vapor. The spot of lipids that eluted with the mobile phase was scraped and dissolved in chloroform. The suspended particles were removed via centrifugation. The liquid sample was subjected to ultra-HPLC-MS/MS. 

The UHPLC-MS/MS equipment employed a C18 chromatography column (2.1 mm × 100 mm; Kinetex, Phenomenex, CA, USA). The injection volume was 5 µL. The mobile phase consisted of solvent A (0.1% formic acid in water) and solvent B (0.1% formic acid in 90% acetonitrile). The column was maintained at 40 °C and the samples were kept at 8 °C. Data were acquired in positive and negative modes. The chromatographic separation period was 23 min for both modes. The mass range for the negative ion mode was 20–1500 *m/z* and for the positive ion mode, it was 20–1300 *m/z*.

## 5. Conclusions

The biomass of *Thraustochytrium* sp. RT2316-16 contained two variants of coenzyme Q, the CoQ_9_ and CoQ_10_. CoQ_9_ occurred in the biomass irrespective of the culture conditions used for growth, suggesting its function as an essential cofactor for the respiratory electron transfer chain. CoQ_10_ in the microbial biomass underwent dramatic changes, especially if the culture conditions favored growth or lipid accumulation. The lack of some B vitamins and *p-*aminobenzoic acid in the culture medium negatively impacted the growth of RT2316-16, but reduced the growth-associated decline in the CoQ_10_ content of the biomass. Depending on the culture conditions, the maximum CoQ_9_ content of the biomass was around 855 μg g^−1^, and the maximum CoQ_10_ content was nearly 10 mg g^−1^. 

## Figures and Tables

**Table 1:** Effect of adding *p*-hydroxybenzoic acid (*p*HB), tyrosine and *p*-aminobenzoic acid (*p*ABA) on CoQ in the biomass of RT2316-16.

Factor	Biomass (g L^−1^)	CoQ_9_ (µg g^−1^)	CoQ_10_ (µg g^−1^)
*p*HB ^£^	3.8 ± 0.5 (72 h)	22 ± 18	ND
*p*HB ^£^	4.9 ± 0.7 (96 h)	3 ± 2	158 ± 19
*p*HB ^¥^	2.4 ± 0.3 (72 h)	4 ± 15	800 ± 12
*p*HB ^¥^	2.4 ± 0.5 (96 h)	2 ± 19	151 ± 3
Control	3.0 ± 0.4 (72 h)	163 ± 39	100 ± 22
No *p*ABA ^†^	1.8 ± 0.3 (72 h)	174 ± 37	489 ± 55

^£^ Composition of the medium (g L^−1^): glucose, 20; yeast extract, 6; monosodium glutamate, 0.6; and the full vitamin solution. Incubation time was 72 h; at 72 h the culture was supplemented with *p*HB to a concentration of 25 mg L^−1^ and incubation was continued for another 24 h (i.e., total incubation of 96 h). ^¥^ Culture medium as in ^£^ with the yeast extract replaced with ammonium sulphate at 5 g L^−1^. ^†^ Culture medium as in ^£^ without *p*ABA.

## Data Availability

The data presented in this study are available on request from the corresponding author.
